# Longitudinal geo-referenced field evidence for the heightened BMI responsiveness of obese women to price discounts on carbonated soft drinks

**DOI:** 10.1371/journal.pone.0261749

**Published:** 2021-12-29

**Authors:** Yun-Hsuan Wu, Spencer Moore, Yu Ma, Laurette Dube

**Affiliations:** 1 Department of Public Health, China Medical University, Taichung, Taiwan; 2 Health & Society Group, Social Sciences Department, Wageningen University & Research, Wageningen, Netherlands; 3 Desautels Faculty of Management, McGill University, Montreal, Quebec, Canada; 4 McGill Centre for the Convergence of Health and Economics, McGill University, Montreal, Quebec, Canada; Medical University of Vienna, AUSTRIA

## Abstract

There is increasing interest in the effect that food environments may have on obesity, particularly through mechanisms related to the marketing and consumption of calorie-dense, nutrient-poor foods and sugary beverages. Price promotions, such as temporary price discounts, have been particularly effective in the marketing of carbonated soft drinks (CSDs) among consumers. Research has also suggested that the purchasing behavior of consumer groups may be differentially sensitive to price discounts on CSDs, with obese women particularly sensitive. In addition, the intensity of price discount in a person’s food environment may also vary across geography and over time. This study examines whether the weight change of obese women, compared to overweight or normal BMI women, is more sensitive to the intensity of price discounts on CSDs in the food environment. This study used longitudinal survey data from 1622 women in the Montreal Neighborhood Networks and Health Aging (MoNNET-HA) Panel. Women were asked to report their height and weight in 2008, 2010 and 2013 in order to calculate women’s BMI in 2008 and their change of weight between 2008 and 2013. Women’s exposure to an unhealthy food environment was based on the frequency in which their neighborhood food stores placed price discounts on CSDs in 2008. The price discount frequency on CSDs within women’s neighborhoods was calculated from Nielsen point-of sales transaction data in 2008 and geocoded to participant’s forward sortation area. The prevalence of obesity and overweight among MoNNET-HA female participants was 18.3% in 2008, 19.9% in 2010 and 20.7% in 2013 respectively. Results showed that among obese women, exposure to unhealthy food environments was associated with a 3.25 kilogram (SE = 1.35, p-value = 0.02) weight gain over the five-year study period. Exposure to price discounts on CSDs may disproportionately affect and reinforce weight gain in women who are already obese.

## Introduction

Obesity has been identified as a serious public health problem and has been described as a global pandemic [1]. Over the past decades, there has been an increased interest in the association between food environments and obesity [2, 3]. Besides the geographical arrangement of food places within local areas, neighborhood food environments also vary in terms of the intensity of marketing efforts to influence food choices, such as food prices and marketing [3]. Within food environments, carbonated soft drinks (CSDs), especially sugary beverages, represent a major source of caloric intake. For example, sugar-sweetened beverages (SSBs) consumption has risen substantially in the past 30 years [4, 5], contributing the largest portion to the caloric intake of national diets [6–8]. SSBs have been associated with weight gain and increased risk of developing obesity among children [5, 9, 10] and adults [9, 11]. Potential explanations for the association among SSBs, increased energy intake, and greater body weight include the added-sugar content, and lower satiety of liquid calories [12].

The Institute of Medicine (IOM) and the World Health Organization (WHO) have both recommended increased governmental action, including tax policies, aimed at reducing SSBs [13, 14]. Policymakers worldwide have responded to these calls for action and numerous countries have recently levied taxes on SSBs. For example, in 2014, Mexico implemented an approximately 10% tax on SSBs (a one peso (0.008 USD) per liter excise tax). The SSB tax resulted in the decreased sale of SSBs and increased sale of water in 2014–2015 compared to the pre-tax period (2007–2013) [15]. Taxing SSBs to reduce consumption relies on the idea of price elasticity, which suggests that consumers respond to higher prices on a product by purchasing less of the product [16, 17]. Research suggests that SSB demand is particularly elastic to price changes [18, 19].

Beyond tax policies, there is also the need to study the impact of other economic dis/incentives on consumer behaviors and outcomes. For example, epidemiological studies have linked the progressive increase in obesity worldwide to the relatively low price of nonessential-energy dense food vis a vis nutrient-rich food [20]. Price discounts impact food purchases and, when discounts are applied to high-caloric food, they may add to a person’s risk of overweight or obesity by reinforcing their preferences for high-caloric food and SSB [2]. By boosting the purchasing of discounted products [21–23], price promotions, including temporary price discounts, are one of the most effective and persuasive tools in marketing. Temporary price discounts heighten consumers’ sense of immediacy to purchase a product, which might result in the increased purchasing and subsequent consumption of those products [21, 23–27]. Previous evidence has linked the marketing practices of price discounts to an obesogenic shift in consumption patterns [21, 28]. Price reductions can lead to a significant increase in consumption [29, 30] through the consumers’ stockpiling of food items, substitution of certain products for others, and/or making unplanned purchases [21, 26]. Price promotions are often applied to obesogenic foods [31–34], especially SSBs [27, 33, 35] because consumers prefer price discounts on unhealthy foods due to guilt-mitigating mechanisms [36, 37]. Consumers feel greater conflict when buying unhealthy food, and price discounts may help justify the purchasing of unhealthy foods by allowing consumers to believe that they are saving money and not overconsuming unhealthy food [37]. There is mounting evidence that such marketing activities contribute to poor dietary intake [21, 38, 39] and rising obesity rates [40–42].

Despite general price elasticity principles, groups may often respond differently to any particular economic dis/incentive [43]. For example, socio-economic status has often been shown to be a key factor influencing a person’s dietary choices and purchasing behavior [44, 45]. Less understood may be the role that gender and physiology play together in influencing purchasing behavior. First, in terms of gender, women, the focus of the present study, may be particularly vulnerable to the influence of food environments on their weight, while also exposing other household members to those environments through the role that they may play in the family. Research has shown, for example, that women often control the bulk of household purchasing decisions for everyday items like groceries and clothing [46, 47]. In the U.S., for example, women account for 85% of all household consumer purchases and 93% of food purchases [47]. Women’s central role in household purchasing and consumption, thus, makes them a prime target for price promotions and other food marketing mechanisms.

Moreover, among women, previous evidence further suggests that obese and overweight individuals, compared to normal BMI controls, may be especially vulnerable to the intensity of SSB price discount because they develop higher reward sensitivity [48]. The association between greater reward sensitivity and obesity may be due to deficiencies in the dopamine signaling, along with associated effects on the brain’s reward systems and a person’s responsiveness to food cues and the environment [49, 50]. In addition, research has shown that food to have a greater reinforcing value in obese compared to non-obese women, suggesting a possible mechanism explaining the higher positive energy balance and food consumption in obese persons [51–54]. Together these various mechanisms may potentially operate to heighten the sensitivity of obese persons to price discounts and the perceived value of such transactions [55–57].

The marketing strategy of price promotion among an obesogenic environment is applied more often to products at SSBs [27, 33, 35] and has been found to be linked to an increased level of food intake [58] which lead to weight gain or even obesity. Yet, to the best of our knowledge, few studies have examined whether price promotion strategies, such as frequent price discounts, might affect women’s weight over time, though the local food environments may target women more directly based on their central role in household purchasing [46]. Since women are more likely to be grocery shoppers and do shop more often [47, 59], they might be easier to stimulate to buy SSBs by price discount and result in increase the risk of being obese, but further work still is needed to better understand the possible link between gender roles in food-related activities, including shopping for food or perceiving food marketing strategies, and obesity. Our present study would like to fill the research gap with women’s weight status vulnerable to local environmental influences. In addition, although previous evidence indicated that overweight or obese individuals may potentially heighten a person’s responsiveness in the local environment [43, 48, 51, 56], no research has used empirical data to examine whether a person’s BMI status might moderate the influence of price discounts on CSDs on personal weight gain. Therefore, expanding upon previous research, we report results of a longitudinal geo-referenced field study to test hypothesizes that obese and overweight women, compared to normal BMI women, would show greater weight gain in response to neighborhood food environment characterized by a greater frequency of price discounts on CSDs.

## Methods

### Sample

The Montreal Neighborhood Networks and Health Aging (MoNNET-HA) Panel provided individual-level data on women’s weight at three-time points, as well as relevant socio-demographic and economic data. The MoNNET-HA used a two-stage stratified cluster sampling design to select a random sample of adults eligible for study participation. In stage one, Montreal Metropolitan Area (MMA) census tracts (N = 862 in 2001 Canada Census) were stratified into tertiles of high, medium, and low household income. One hundred census tracts were selected from each tertile (n = 300). In stage two, households were selected at random in each census tract until a quota of three adults were interviewed in each of three age stratums: 25–44 years old, 45–64, and 65 or older. This resulted in a total of 9 respondents per tract, except for seven tracts in which four participants were interviewed, and a final sample size of 2707 adult nested in 161 forward sortation areas (FSAs). To be eligible for the study, participants had to be (1) be non-institutionalized, (2) have resided at their current address for at least one year, and (3) able to complete the questionnaire in French or English. Ethics approval for the MoNNET-HA was awarded by the Committee of Scientific Evaluation and Research Ethics of the Centre de Recherche at the Centre Hospitalier de l’Université de Montréal (CHUM) (#ND07.049) and the General Research Ethics Board of Queen’s University (GPHE-148-13) [60]. Verbal consent was obtained from each participants at the beginning of the survey administration process and was documented electronically on the CATI system. For this study, we selected and extracted female participants from the MoNNET-HA Panel.

We measured the food environment using the Nielsen Retail Measurement data. Point-of-sales transaction (PoST) data for carbonated soft drinks (CSDs) was purchased from the Nielsen Corporation for a large Canadian province. The PoST dataset covers retail grocery sales in grocery stores, mass merchandisers, and convenience stores, capturing approximately 72% of the total grocery sales in this province. The PoST dataset was geo-located using forward sortation areas (FSA). The FSA is a geographically-defined administrative unit consisting of the first 3 characters of the 6-character Canadian postal code and containing 8,000 households on average [61]. We are able to link the Nielsen PoST data to the MoNNET-HA Panel by FSA. Relevant marketing data for this analysis consisted of weekly price and price discount at stock keeping unit (SKU; unique identifier for each distinct product sold in a store).

### Measurement

#### Outcome–weight in 2008, 2010 and 2013

The MoNNET-HA Panel participants reported their weight in kilograms in 2008, 2010 and 2013. To account for possible biases, an adjusted weight was calculated using a correction factor that Statistics Canada developed from the analysis of self-reported weight and BMI data [62].

#### Main exposure variables–CSDs price discount frequency

In this study, price promotions on CSDs were measured according to the frequency of price discounts on CSDs in 2008. These measures have been described in detail elsewhere [23]. In brief, only products coded as “Carbonated Soft Drinks (CSDs)”, which included all SKUs for flavored soft drinks containing sugar (but not sugar-free diet soda items), were extracted from the Nielsen PoST data. Price discounts on CSDs were defined as the number of weeks in which the price of CSDs was at least two standard deviations below its average price [23, 63]. This store-level information was then aggregated to the FSA-level to represent neighborhood-level exposure to price discounts on CSDs.

#### Other variables

Women’s age category, marital status, household language, socioeconomic status (SES) and smoking status were taken from wave one of the MoNNET-HA Panel and considered fixed variables for these analyses. Participants self-identified their gender and their ages were grouped into six categories: (1) 25–34, (2) 35–44, (3) 45–54, (4) 55–64, (5) 65–74, and (6) 75 years or more. Participants were also asked to indicate their marital status as: (1) currently married or in a common-law relationship, (2) single, or (3) formerly married, which included separated, divorced, or widowed. Respondents’ primary household language was grouped into French, English and other. Participants’ SES was a composite score based on a principal components analysis (PCA) of their educational attainment, household income, and employment status [64]. Participants also were asked to indicate whether they had or had not smoked in the last 30 days. The “wave” variable was included to mark the time period in which the obesity status was assessed. The MoNNET-HA Panel participants reported their height in meters and weight in kilograms at baseline and this information was used to estimate the self-reported baseline weight and BMI status (kg/m^2^). To account for possible reporting biases, an adjusted BMI was calculated using a correction factor that Statistics Canada developed from the analysis of self-reported BMI data [62]. In addition, we used the centering baseline BMI, which subtracted the mean from participants’ BMI, as one of the covariates adjusted in the model. In the stratified analyses, we stratified our participants based on their baseline weight status, which we used body mass index (BMI) as a tool to classify the weight status into three categories: normal weight as 18.5 kg/m^2^ ≤ BMI < 25 kg/ m^2^; overweight as 25 kg/ m^2^ ≤ BMI < 30 kg/ m^2^, and obesity as BMI ≥ 30 kg/ m^2^. To adjust for potential area-level confounders of the relationship between FSA-level CSDs price discount frequency and individual obesity, we included census-tract level 2006 Canada census information on population density and socioeconomic status.

### Analyses

Multilevel linear regression modeling was used to examine whether the frequency of CSDs price discounts was associated with individuals’ weight over the five-year study period and whether this association was modified by women’s baseline weight status. A multi-stage model-building process was undertaken. Model one examined the simple relationship between CSDs price discounts and individuals’ weight over time. Model two additionally adjusted for age, marital status, SES, smoking status, wave, centering baseline BMI and area-level socioeconomic conditions and population density in the model. Model 3 added the interaction term between individuals’ centering baseline BMI and neighborhood exposure to price discounts to test whether individuals’ baseline BMI modified the influence of neighborhood exposure to price discounts on individual weight. Finally, based on findings, we stratified our participants into obese, overweight, and normal-weight women to examine the association between exposure to CSDs price discounts and women’s weight. Multilevel regression was used, with repeated measures of weight nested in women and women nested in the FSA in which they resided. Regression coefficients and 95% confidence intervals are reported. All analyses were carried out with STATA using gllamm, version 14 (Stata, College Station, TX, USA).

## Results

After excluding participants with missing data, the final sample size consisted of 1,622 women. The prevalence of obesity among the MoNNET-HA female participants was 18.3% in 2008, 19.9% in 2010 and 20.7% in 2013. Table 1 provides baseline descriptive information on the study sample. Generally, participants with obesity at baseline tended to be older, single, unemployed, and have lower levels of income and educational attainment.

**Table 1:** Characteristics of female participants in the Montreal Neighborhood Networks and Healthy Aging (MoNNET-HA) in 2008 (at the baseline, n = 1,622; the prevalence of obesity: 18.3% in 2008, 19.9% in 2010 and 20.7% in 2013).

		Normal at baseline		Overweight at baseline		Obesity at baseline	
		n = 805		n = 521		n = 296	
		%		%		%	
Age							
	25–34	19.3		10.6		11.2	
	35–44	19.9		12.3		13.9	
	45–54	18.6		19.0		22.3	
	55–64	14.9		19.0		15.5	
	65–74	17.0		25.7		27.0	
	75+	10.3		13.4		10.1	
Marriage							
	Married/Common Law	53.7		53.8		47.3	
	Single	18.2		15.5		23.0	
	Have Married	28.1		30.8		29.7	
Education							
	No degree	10.1		16.8		18.4	
	High school/Trade	24.6		30.6		42.9	
	College	22.6		20.5		17.7	
	University	42.7		32.1		21.1	
Employed							
	No	41.9		56.2		56.8	
	Yes	58.1		43.8		43.2	
Income							
	<28,000	19.0		24.6		32.8	
	28,000–49,000	27.7		28.4		29.1	
	50,000–74,000	27.1		28.6		23.7	
	75,000–100,000	12.6		11.1		9.8	
	>100,000	13.7		7.3		4.7	
Smoking							
	No	75.4		82.0		79.4	
	Yes	24.6		18.0		20.6	
		**Mean**	**SD**	**Mean**	**SD**	**Mean**	**SD**
CSDs price discount frequency		0.29	0.24	0.29	0.23	0.31	0.24
Census track SES		0.06	0.90	0.003	0.88	-0.084	0.93
Census track density		16.08	5.95	16.09	5.32	15.99	5.66

Table 2 shows the results from the multilevel linear regression of women’s weight on the frequency of CSDs price discounts. In model 2, participants with higher baseline weight status gained more weight over the five-year study period (β = 2.22, SE = 0.04, P < 0.001). Meanwhile, participants who were aged 65 and older (65–74: β = -2.97, SE = 0.83, P = < 0.001; 75+: β = -4.47, SE = 0.99, P = < 0.001), had other language as their primary language at home (β = -1.68, SE = 0.78, P = 0.03) and were smoking (β = -1.10, SE = 0.55, P = 0.05) tended to lose weight over the five-year period. Results showed that neighborhood exposure to price discounts was not directly associated with changes in women’s weight over the same period (β = -0.84, SE = 0.97, P = 0.39). Model 3 showed that women’s centering baseline BMI modified the relationship between neighborhood exposure to CSD price discounts and women’s’ weight change (β = -0.52, SE = 0.16, P = 0.002). To examine this effect modification, we stratified our analyses by baseline BMI categories (i.e., normal, overweight and obesity) with results shown in Table 3. Among women who were obese at baseline, greater exposure to neighborhood price discounts on CSDs led to increased weight gain over the five-year study period (β = 3.25, SE = 1.35, p-value = 0.02). Among women who were normal or overweight at baseline, there was no relationship between greater exposure to neighborhood price discounts on CSDs and changes in weight (normal at baseline: β = -0.15, SE = 0.85, p-value = 0.86; overweight at baseline: β = -0.30, SE = 0.70, p-value = 0.67).

**Table 2:** Results of regression analyses examining the association between Carbonated Soft Drinks (CSDs) price discount frequency and women’s weight (kg), n = 1,622.

		Model 1			Model 2			Model 3		
		β	SE	P-value	β	SE	P-value	β	SE	P-value
**Fixed effect**										
CSDs price discount frequency		0.56	1.65	0.73	-0.84	0.97	0.39	-0.87	0.97	0.37
Age										
	25–34				(reference)			(reference)		
	35–44				0.63	0.78	0.43	0.50	0.78	0.53
	45–54				-1.24	0.76	0.11	-1.41	0.76	0.07
	55–64				-1.41	0.81	0.08	-1.51	0.80	0.06
	65–74				-2.97	0.83	< 0.001	-3.00	0.83	< 0.001
	75+				-4.47	0.99	< 0.001	-4.54	0.99	< 0.001
Marital status										
	Married/Common Law				(reference)			(reference)		
	Single				0.31	0.59	0.60	0.25	0.59	0.67
	Have Married				-0.58	0.50	0.25	-0.56	0.50	0.26
Household language										
	French				(reference)			(reference)		
	English				0.50	0.67	0.45	0.59	0.66	0.38
	Other				-1.68	0.78	0.03	-1.67	0.78	0.03
Socioeconomic status					-0.05	0.35	0.89	0.01	0.35	0.97
Smoking										
	No				(reference)			(reference)		
	Yes				-1.10	0.55	0.05	-1.03	0.55	0.06
Wave					0.76	0.19	< 0.001	0.76	0.19	< 0.001
Centering baseline BMI					2.22	0.04	< 0.001	2.38	0.07	< 0.001
Census track population density					0.01	0.05	0.92	0.01	0.05	0.83
Census track SES					0.13	0.34	0.71	0.14	0.34	0.67
CSDs price discount frequency * Centering baseline BMI								-0.52	0.16	0.002
Intercept		68.44	0.62	< 0.001	11.80	1.56	< 0.001	7.48	2.08	< 0.001
		**Variance**			**Variance**			**Variance**		
**Random effect**										
Level 2		170.06			33.56			33.13		
Level 3		0.00			1.65			1.70		

**Table 3:** Results of regression analyses examined the relationship between CSDs price discount frequency and women’s weights, stratified by baseline weight status*, n = 1,622.

Baseline weight status	β	SE	P-value
Normal at baseline	-0.15	0.82	0.86
Overweight at baseline	-0.30	0.70	0.67
Obesity at baseline	3.25	1.35	0.02

*Note: Estimates adjusted age, marital status, household language, socioeconomic status smoking, wave, centering baseline weights, census track population density and census track SES

## Discussion

This study showed that, while no significant relationship emerged at the population level between long-term weight change and price discount intensity, obese women whose neighborhood environment had more frequent price discounts on CSDs increased their weight over a five-year period. Compared to overweight or normal weight women, women who are obese may be differentially responsive to price discounts and thus at greater risk of weight gain over time. Various mechanisms–social, psychological, and neurological—might explain obese women’s responsiveness to price promotion strategies. First, women often have the predominant role in household food purchases [46, 47] and they also spend longer hours on food purchasing [59]. Evidence has also suggested that women may be potentially more sensitive to food cues in the local retail environment. For example, cross-sectional and longitudinal research in children showed that the presence of food outlets, including fast-food restaurants and convenience stores, was associated with weight status and weight change in girls but not boys [65, 66]. Meanwhile, other studies indicated that the amount of money spent on food-away-from-home was associated with the body mass index (BMI) of older females but not older males [67]. Since women are largely responsible for the preparation of food in a household and may be susceptible to the food environment, women may be at heightened sensitivity to price promotion programs and the perceived value of price discounts.

Second, neurobehavioral mechanisms such as reward sensitivity may operate to heighten a person’s responsiveness to food cues in the local environment. Reward sensitivity as a psychobiological characteristic mainly regulates via dopamine pathways in the brain, with increased reward sensitivity linked to eating behaviors and obesity due to the higher preference for highly palatable food (i.e. sweet or fat food) [68] and greater response to high-calorie cues in the local environment [43]. Not only has reward sensitivity been shown positively associated with obesity [69] but previous research has also shown reward sensitivity contributing to weight gain in women [68, 70–73]. According to Mobbs et al. (2010), high sensitivity to reward in overweight and obese women may relate to a dysregulation in dopamine signaling, akin to addiction. Dopamine signaling and its behavioral and body weight consequence are doubly affected by the response to the person obesity status and the superior food reinforcement tied to SSB sugar content [51, 52].

Food reinforcement, which refers to stimulus and response associations related to food acquisition as well as intake [51–54] is also regulated by the level of dopamine activation [51, 68]. Such reinforcement may also have a different influence on individuals due to their weight status. Obese individuals often find food more reinforcing than healthy weight individuals [51, 52] and may thus be more motivated to work harder to earn [51, 74, 75] or consume food [51, 52, 74]. Moreover, food and food-related cues in the in-store retail environment, such as price discount/promotion, could also stimulate the dopamine system to activate reward-related brain circuits [76, 77] and motivate eating behaviors through heighten a person’s sensitivity and reinforcing value, especially on obese subjects [56, 74]. This line of work further shows that obese subjects who respond more intensely to food reinforcement also tend to consume more energy-dense foods than normal-weight ones with low food reinforcement [51, 53, 54].

Finally, food environments may differentially affect the expression of these social, psychological, and neurobehavioral mechanisms for obese vs normal weight individuals. For example, compared to normal-weight individuals, obese persons report a stronger motivation to eat when exposed to food cues and a tendency to attend selectively to high caloric food [57]. Attention bias for high caloric food may lead obese individuals to be more vulnerable to food marketing strategies on products high in fat, sugar and/or salt. Experimental neuroscience research has further shown that responsiveness to differential attentional salience to sensory-functional attributes for natural vs transformed food was modulated by a person’s BMI, with sensory attribute of transformed food being particularly salient for obese, while functional attribute of natural food being particularly salient for lower BMI individuals [78]. Thus, enhanced food cue-reactivity and attention bias among obese women in the context of frequent price discounts may play an important role in the purchasing and consumption of surgery beverages and CSDs. Further research need to explore how theses various mechanisms related to high responsiveness to price promotion strategies among obese women may lead weight gain as a result of overconsumption of sugar [79]. Evidence from previous large cross-sectional and cohort studies showed that increased intake of SSBs, where are the primary source of added sugar [80, 81], would positively associated with weight gain as well as obesity in both children and adults [82, 83]. SSBs commonly lack nutritional value as well as satiety signals and represent excess energy in the daily diet and, so people tend not to offset their calories by reducing their consumption of other food or drink [84, 85], resulting in an increase in calorie intake and contributing to overweight and obesity [86].

There were a number of study limitations to note. First, our measure of the food environment was based on women’s exposure to price discounts in their local food and retail stores. Yet, women may not only shop in their neighborhood—they may also shop outside their place of residence (e.g., near their work place) or online. Our study does not capture these non-residential food environments. Second, this study assumes that neighborhood CSD price discounts represent a generalized exposure for all women, influencing women’s risk of obesity through their impact on women’s purchasing of CSDs. Our study lacks the individual-level data to assess the relationship between exposure and actual purchasing and consumption of CSDs. Third, in terms of the food-marketing environment, our study focuses on only one marketing strategy. However, there are other marketing strategies that may individually or jointly impact food choice [28]. Future research might investigate how diverse dimensions of the local food marketing environment might influence food purchasing and consumption.

Despite these limitations, the current study represents a novel assessment of the relationship between neighborhood price discounts and women’s risk of obesity using longitudinal data. Price discount strategies are a central marketing strategy in the sale of healthy and non-healthy foods and beverages. Since the demand of carbonated soft drinks (CSDs) is particularly elastic to price changes, price discounts have been particularly effective in boosting the sales of CSDs. Yet, not all individuals exposed to obesogenic food environments will necessarily become obese. Therefore, understanding which factors might influence individual responsiveness to environmental food cues can help the development and implementation of interventions to address obesity. Our study can thus inform the design of specific interventions that might limit price promotion on CSDs in general.
